# An analysis of English national policy approaches to health inequalities: ‘transforming children and young people’s mental health provision’ and its consultation process

**DOI:** 10.1186/s12889-022-13473-6

**Published:** 2022-05-31

**Authors:** Naomi Griffin, Jonathan Wistow, Hannah Fairbrother, Eleanor Holding, Mihirini Sirisena, Katie Powell, Carolyn Summerbell

**Affiliations:** 1 Durham University, Durham, UK; 2 Fuse, The UK Centre for Translational Research in Public Health, Newcastle, UK; 3 University of Sheffield, Sheffield, UK; 4 ScHARR, School of Health and Related Research, University of Sheffield, Sheffield, UK; 5 Newcastle University, Newcastle, UK

**Keywords:** Health inequalities, Children, Mental health, National Policy, Narrative review

## Abstract

**Background:**

A national policy for England, published in 2017, entitled ‘Transforming Children and Young People’s Mental Health Provision’ aimed to address the increasing prevalence mental health problems in children and tackle inequalities. In the context of this policy’s implementation as ongoing and the effects of the Covid-19 pandemic, the need for appropriate, timely and ongoing national government commitment is vital.

**Methods:**

A narrative review using a problem representation evaluation [1], we critiqued the policy and related consultation documents using a social determinants of health perspective. We also reviewed wider policy discourses through engaging with stakeholder responses, providing an innovative methodological contribution to scholarship on public health policy and health inequalities.

**Results:**

We found absences and oversights in relation to inequalities (most notably the lack of acknowledgement that mental health can cause inequalities), access, workforce capacity, and the impacts of cuts and austerity on service provision. We suggest these inadequacies may have been avoided if stakeholder responses to the consultation process had been more meaningfully addressed. We illustrate how ‘problems’ are discursively created through the process of policy development, justified using specific types of evidence, and that this process is politically motivated. Local policy makers have a critical role in translating and adapting national policy for their communities but are constrained by absences and oversights in relation to health inequalities.

**Conclusions:**

This narrative review illustrates how policy discourse frames and produces ‘problems’, and how the evidence used is selected and justified politically. This review contributes to the existing transdisciplinary field of knowledge about how using methods from political and social science disciplines can reveal new insights when critiquing and influencing policy approaches to health inequalities.

**Supplementary Information:**

The online version contains supplementary material available at 10.1186/s12889-022-13473-6.

## Key messages

- This health policy illustrates a narrow framing of the links between inequalities and mental health, revealing gaps between claims about priorities and policy changes proposed.
- Stakeholders identified this limited framing in the consultation process, but were largely ignored.
- A policy narrative, politically wedded to austerity, was used to frame the problem and select the evidence. This narrative was made evident by engaging with wider discourses and critically examining problem representation.
- Using methods from political and social science disciplines reveal new insights about health policies.

## Background

In 2017 the Department of Health and Social Care and the Department of Education for England (UK) released the long-awaited ‘Transforming Children and Young People’s Mental Health Provision: A Green Paper’ [2]. The Green Paper was published when Theresa May (Conservative Government) was Prime minister and was presented as a follow-on from the Coalition Government’s Future in Mind [3]. The Green Paper aimed to address the rising prevalence of mental health problems in children and young people (or CYP) and the inequalities in life chances faced by children who experience mental health problems. In 2021 the Mental Health Foundation reported that approximately one in six children are affected by mental health issues in the UK, yet 75% of those affected have not received sufficient support and intervention at an early age [4]. Approximately half of all mental health conditions first occur for individuals by age 14, and mental health problems are known to persist into adulthood (Education and Health and Social Care Committees [5]. The Care Quality Commission [6] has highlighted concerns about children and young people’s experiences of mental health services, noting inconsistencies in quality and accessibility and extremely lengthy waiting times in some areas. They emphasise that inadequate support or poor experiences of mental health services create further barriers for many children. With rates of mental health disorders rising among children and young people, from one in nine 5–16 year olds in 2017 to one in six in June 2020 [7], and increasing concern about the impact of the Covid-19 pandemic on mental health (particularly in relation to health inequalities, though more data in needed) [8], inadequate support for the mental health of children and young people is likely an increasingly worsening problem.

Using the Green Paper, and its consultation process, as an ‘entry point’ [9] into the child health system, we draw from different actors and evidence bases within a social determinants of health perspective [10], analysing how external stakeholders and experts responded to the policy and how it addresses the growing mental health crisis among children and young people, particularly in relation to inequalities. We used Bacchi’s [1] ‘What’s the Problem Represented to be?’ (WPR) approach to critique policies as ‘political interventions’ by analysing what is produced through particular problem representations and thus how policy pathways are justified and embedded.

This paper aims to develop understanding of how health inequality is conceptualised in national policy in order to identify effective pathways to reducing inequalities among children and young people. In so doing, we develop a theoretical understanding of the pathways through which local programmes and interventions are expected to impact on outcomes of interest, within the context of the fluid and adaptive nature of health systems. The paper aims to aid understanding of the political agendas to which policy might be tied, the historical context for particular policies and the evolving public discussion around developing policy areas. This paper speaks to the fields of child equity and health, social policy, social determinants of health perspectives (notably how they must be embedded fully within policy processes), mental health and inequality, policy pathways and implementation, policy discourses, and the power of discourse in progressing ideological decision making.

### Mental health and inequalities in England

It has long been established that health is socially and politically produced (see for example: [11–18]. Poverty and social disadvantage are linked to the prevalence of mental health problems as both contributing causal factors and consequences of mental ill-health [19, 20]. Children living in the poorest households in England are approximately three times more likely to experience mental health problems and the health gap between the most and least deprived is growing [17]. There are a number of other factors exacerbating health inequalities in mental health including: stigma associated with mental health problems, poor housing and living conditions, lack of security in housing, being part of a marginalised group (such as asylum seekers and refugee children who are more likely to experience trauma which require tailored intervention and support), and vulnerable individuals living in marginalised communities [19]. The CQC ([6], p7) identified statistics that show higher prevalence of mental health problems in England for ‘*looked after children, care leavers, young people in the criminal justice system, lesbian, gay, bisexual and trans [gender] children and young people, and those with physical disabilities or learning disabilities*.’

### Social determinants of health

A ‘social determinants of health’ perspective explores how individual experience of health is intwined with micro and macro social and political contexts, which lead to health inequalities [15, 21]. In England, the development of a social determinants of health perspective designed to shape policy culminated in the 2010 Marmot Review [16], which emphasised the significance of the ‘causes of the causes’ of health outcomes. The review focused on; early years; education; work; income; and communities, as areas in which the social gradient in health was particularly evident with persistent and complex causes and relationships cutting across inequalities ([16], p.84). In a report considering the Marmot Review 10 years on, Marmot et al., ([17], p5) argue, ‘health is closely linked to the conditions in which people are born, grow, live, work and age and inequities in power, money and resources – the social determinants of health.’ Bambra et al. [22] illustrate the significance of place in understanding the macro and micro political economy and social influences on health. Marmot et al., ([17], p140) conclude that ‘system-wide approaches based on cross-sector partnerships are a prerequisite for effective action on the social determinants and health inequalities.’ Social determinants of health approaches, therefore, sit comfortably with Walby et al.’s [23] application of critical realism to social systems as interlinked levels, emerging from, but not reducible to, each other, thereby not reducing analysis to the micro level of agency or the macro level of structure – both take their place in the analysis. At the macro-level, however, there are concerns about how both policy and research neglect the structural forces (such as class) that are key causal factors in producing social and economic inequalities and health inequalities (see, for example, [12, 14, 15, 18, 21, 24–27]. Despite the evidence, there remains a lack of engagement with this knowledge within policy networks and contexts [15, 28, 29]. Where policy and policy networks do acknowledge the social determinants of health, the politics of power that creates and sustains inequalities and determines health disparities go largely unacknowledged [15, 17].

### Mental health policy context and the social determinants of health

The absences within policy of engagement with the social determinants of health worsened over the past decade through a shift in policy discourse towards a focus on behaviour change and individualism [2, 15, 29–31] possibly most notably in mental health policy discourse since 2010 [28]. Kriznik et al. [29] noted a growth in recognition of multiple determinants of health between 1976 and 2010 but this was not translated into actual policy plans, interventions or evaluation plans. Callaghan et al. [28] describe a gradual shift in policy over the past two decades towards a medical model understanding of mental health that focuses more on the biological characteristics and individual determinants of mental health. Callaghan et al. [28] also note a shift in mental health policy from strategies to achieve good mental health for all and enabling people to ‘reach their potential’ to policies which are more focused on mental health as illness, aiming to help people ‘get by’. A focus on the individual and illness in policy obscures power dynamics (between groups and institutions) and limits engagement with social context, which works to minimise the social determinants of health [29].

The change in government in 2010 to a Conservative and Liberal Democrat coalition and the introduction of an austerity programme have been identified as a significant change in direction [19, 28, 29]. For Callaghan et al. ([28], p111) ‘the shifts in mental health policy before and after this point in time are dramatic, reflecting both changes in government ideology, and in the socio-economic context in which policies are produced and embedded.’ For example, the NHS reported that between 2010 and 2013 £50million was cut from child and adolescent mental health services (CAMHS) budgets [28]. The first Marmot review [16] showed that the localisation of health spending pre 2010 had already resulted in areas with the highest levels of socio-economic deprivation receiving lower budget allocations relative to need. Subsequently, further reduction of funding linked to local authority deprivation levels has led to the areas with the highest levels of deprivation (and therefore need) being hit hardest by cuts, increasing geographical inequalities [19]. Whilst some areas improved their performance in terms of geographical health inequalities under New Labour’s health inequalities strategy [31] inequalities remained largely persistent [16, 17]. Robinson et al., [31] and Marmot et al. [17] have highlighted the negative impact of austerity on health inequalities and Callaghan et al., [28], Cummins [19], and Kriznik et al., [29] suggest it has also increased the likelihood of mental health problems associated with social disadvantage.

Researchers and stakeholders have highlighted the barriers to mental health support faced by childran and young people, which have worsened due to austerity [16, 17, 19, 20, 32]. In particular, the high threshold for referral to CAMHS has resulted in GPs rejecting the majority of referrals in some places, which discourages young people from seeking further support [32]. Mental health problems that go untreated are likely to extend into adulthood [16, 17] and therefore the gaps and problems of inequality in mental health service access and provision must be addressed.

The impact of Covid-19 pandemic on the mental health of children and on mental health services will likely further exacerbate inequalities going forward [8]. A UK parliamentary briefing in July 2020 [33] highlighted the increased need for mental health support for children and young people as a result of the stresses caused by the Covid-19 pandemic (though the interaction between these stressors and already established inequalities is not covered). The May 2021 report by the CMH stated that over the next 3–5 years, approximately 1.5 million children and young people in England will need mental health support as a ‘direct result’ of the Covid-19 pandemic. In March 2021 the UK government released the ‘COVID-19 mental health and wellbeing recovery action plan’ [34] which commits a £15 million investment in preventing mental health problems for the most disadvantaged local authorities. However, the plans are generally limited to 2021 and 2022 and the scope is limited to predominantly health and social services [8, 35], where a wider scope and longer-term plan is needed to respond to the implications of the pandemic, and to continue the implementation the Green paper in light of said implications [8, 36].

In response to the growing prevalence of mental health problems and inequalities among children and young people in the context of changes in policy and service provision in recent years and the impact of the Covid-19 pandemic, we agree that ‘transforming’ mental health provision for children and young people is imperative. Our approach to policy analysis aims to highlight mechanisms that are used in the framing of said ‘transformation’ that showcase the nature and extent of the response in terms of policy pathways to reducing health inequalities.

## Methodology

The aim of this research was to investigate the narratives embedded within policy documents and discourses, and the responses to them, in order to consider the ways in which policy narratives themselves lead to particular outcomes. In seeking to understand policy responses to health inequalities we consider these to be both complex and to cut across systems (see, for example, [18, 25, 37, 38]. The overarching research question was ‘how does the way policy problems are framed lead to particular policy responses in relation to policies on CYP mental health and inequalities?’ In so doing, we intend to contribute to debates about the politics of both framing and understanding health inequalities within and through policy discourses [14]. The objectives of this research were a) to identify gaps (absences) in relation to inequalities in the policy and related consultation documents using a social determinants of health perspective b) to identify where such gaps were highlighted in stakeholder responses but ignored. Rutter et al., ([38], p2602) argue for a ‘complex systems model of public health [that] conceptualises poor health and health inequalities as outcomes of a multitude of interdependent elements within a connected whole.’ It is important to recognise that adopting a complex systems approach requires a pragmatic approach to research and there will be much going on ‘outside the field of vision of an individual study’ ([39], p37). In this respect, Wistow et al. [18] contend that if we accept that health inequalities are complex then we must also accept that our knowledge is often imperfect and uncertain and, therefore, requires collaboration between different types of knowledge. Head’s [40] ‘three lenses of evidence-based policy’ is instructive here and includes: political knowledge; scientific (research-based) knowledge; and practical implementation knowledge. Head [40] concludes that the three lenses may suggest divergent perspectives on whether and how to increase mutual understanding and shared perspectives. Here, we follow Jessop’s [9] approach of using policy areas and policies as specific ‘entry points’ to identify and understand divergent perspectives about the role of the state and associated organisations and actors in considering pathways to change in reducing inequalities in child health [41]. In other words, investigating policies and how different types of evidentiary bases come together in response to these, can be used to enter, and explore, the complex systems the policies themselves are seeking to influence and change.

Our narrative review used Bacchi’s [1] ‘What’s the Problem Represented to be?’ (WPR) approach to analyse the Green Paper on Transforming Children and Young People’s Mental Health [2]. First, we identified proposed pathways from national policies expected to lead to reductions in inequalities in child health, i.e. the programme theory of the policy and in so doing identify, ‘underlying assumptions about how an intervention is meant to work and what impacts it is expected to have.’ This process was informed by Pawson et al.’s ([42], p.21) ‘first-step’ in their model of research synthesis which is designed to work with complex social interventions or programmes, and which is based on the emerging ‘realist’ approach to evaluation. We then used Bacchi’s [1] WPR approach to dissect taken for granted ideas within government and external discourses to analyse how ‘problems’ are discursively created within policy documents through ‘problem representations’; our focus was on narratives about how policies are conceptualised. We use the Green Paper as an ‘entry point’ into the government’s and key stakeholders’ understanding of health inequalities. Table 1 provides an overview of the criteria we used to extract data from both the Green Paper and the policy discourse, across a range of interests and stakeholders, arising from this. In considering discourses about the policy, the inherently political and value-based nature of policy debate and decision-making comes into play, which leads Head ([40], p.9) to conclude that policy decisions are, ‘not deduced primarily from facts and empirical models, but from politics, judgement and debate’. The goal here is not necessarily to identify some underlying causal model, as much as it is about exploring and learning how various interventions or strategies might play out for a given policy and the larger complex system in which it is situated (after, [43].

**Table 1:** Data Extraction Criteria

Problem representation [1]	
• **What is the ‘problem’ represented to be?** o How does the policy represent the problem under investigation? o How has this representation come about? o ‘The problem’ can be inferred from the proposed ‘solution’.	
• **Assumptions underpinning the representation of the problem** o What are the presuppositions/ assumptions that underlie the representation and its concepts and categories? • **Effects of problem representation** o What effects are produced by this representation of the ‘problem’?	
• **What is left unproblematic and how might the policy response differ** o What is left unproblematic in this problem representation? Where are the silences? o Can the ‘problem’ be conceptualized differently? o What is left out of the problem representation?	

### Data extraction and analysis

The inclusion criteria for the policy extractions was: national policies published between 2013 and 2019 which explicitly have a focus on children and young people’s mental health which also explicitly aimed to reduce health inequalities in children and young people (relating to England, excluding policies relating to Northern Ireland, Scotland and Wales). A coding framework was developed, using the questions in Table 1, to extract data from policy documents. We then extracted responses using online Google searches (set within a month of key release dates) to capture a range of responses that would be publicly available around key dates in the policy making process, related to the coding framework developed. We completed a search either when we got to the end of the search results or once five pages of non-relevant (not related directly to the green paper) searches were met. We also looked at official responses (via the Parliament website) to the consultation process. Once all documents and responses were coded, we used thematic analysis to pull out core themes that cut across the policy documents and responses, as well as highlighting absences highlighted by the inclusion of responses broader than the policy documents themselves. The key policy document selected (the Green Paper) was independently double data extracted by researchers with different academic backgrounds (NG and MS) to allow more diverse discussion of the findings [44].

Table S1 (see supplementary file) shows the results of four Google searches carried out in response to a. the release of ‘Transforming Children and Young People’s Mental Health Provision Green Paper [2] b. the consultation deadline (2nd March 2018) c. the release of ‘The Government’s Green Paper on mental health: failing a generation report’ [5] and d. the release of the Government response to consultation & release of Government response to the ‘failing a generation report’ [45, 46]. Simple descriptions (without reflection) have been excluded from the table, as have responses not directly related to the Green Paper and subsequent documents or responses not included in findings and analysis in this paper. In the findings and analysis section we refer to the stakeholder responses by number, corresponding to the table.

## Findings and analysis

Our findings and analysis are split into two sections: the first explores ‘problem representation’ and the pathways to anticipated changes in the policy, then we explore assumptions and effects of the problem representation.

### Programme theory and problem representation

*Transforming Children and Young People’s Mental Health Provision: a Green Paper* ([2] , p.3) states:

*‘This green paper … sets out our ambition to go further to ensure that children and young people showing early signs of distress are always able to access the right help, in the right setting, when they need it.’*

The ‘programme theory’ designed to achieve this ambition is developed across three main proposals that seek to improve access to early intervention for children and young people with mild-moderate mental health problems, in order to both reduce waiting times and the impact that mental health problems in childhood have on later life. The policy emphasises the additional cost of children and young people seeking medical intervention for mental health concerns from specialist NHS services and the longer term societal financial cost implications of children and young people struggling with mental health problems as key motivations for the policy. According to the Green Paper, the longer term costs of mental ill-health in childhood include: unemployment and becoming recipients of welfare benefits as adults, links between conduct disorders in young people and criminal activity in adulthood, higher costs to public services where mental health problems have progressed, and increased likelihood of lost working days due to stress, depression and anxiety [2]. This focus may imply that saving public money is emphasised more strongly than a desire to reduce inequalities.

The first proposal sought to encourage all schools, through training ‘incentives’, to appoint a Designated Senior Lead for Mental Health. The Green Paper considers schools to be an ideal site for early (non-clinical) intervention and support, highlighting that some schools and colleges already have systems in place to successfully address this need [2]. The ‘problem’ in Bacchi’s [1] sense is thus represented to be a lack of support in schools for early intervention leading to greater pressure on NHS specialist services (i.e. implying inappropriate referrals of patients that would be better responded to in school settings, rather than a need for greater funding or capacity within NHS specialist support to respond to need). The premise explained was that parents and wider communities look to schools and colleges for help and advice about responding to children and young people’s mental health needs and so Designated Senior Leads also have the potential to be instrumental in supporting children outside of schools [2]. The policy pathway implies that greater support in schools will result in less reliance on specialist NHS support.

The second proposal was to introduce Mental Health Support Teams to work with and between schools, colleges and the NHS, supervised by NHS mental health professionals.

‘*we anticipate that, in the long term, the creation of the new Mental Health Support Teams will lead to a reduction in referrals to NHS services, as earlier intervention prevents problems escalating*.’ ([2], p23)

As a result, it is expected that requests for specialist services would reduce, resulting in improved provision of specialist services in terms of efficiency and reduced waiting time for those who need it most [2].

The third proposal was the introduction of a four-week waiting time trial for access to specialist NHS child and adolescent mental health services. The national average for waiting times at the time that the Green Paper was published was about 12 weeks but with significant geographic inequalities, for example, one provider has an average waiting time of 100 weeks between referral and treatment commencement [2]. The introduction of mental health support teams and Designated Senior Leads is proposed to alleviate pressure on medical services, and therefore reducing inequalities in waiting times [2]. The need to reduce pressure on services is presented in the Green Paper more as the result of inadequate joined-up working, rather than a problem related to socio-economic characteristics of place or lack of service delivery capacity. The aim to reduce waiting times by alleviating the number of NHS referrals presents the problem not as one that is based in place specificities or a lack of capacity but rather as a lack of joined-up working (discussed further in Section 2) and inappropriate referrals.

### Assumptions and effects of problem representation

Here we extend the analysis of the Green Paper by focusing on what assumptions are made, and what potential effects are produced by the problem representation and programme theory outlined in section 1, and by drawing on responses to the Green Paper (Table S1). A number of factors were identified as negatively impacting the capacity of health systems to respond to children and young people’s mental health problems. These include: ‘austerity’ (Table S1:35) or ‘government cuts’ to Local Authority budgets, MH services, social services, health visitors and school nurses (Table S1:18, 26, 27, 32, 35, 36, 37, 39, 43, 44, 49, 52) and cuts to school budgets reducing capacity to provide MH support through counselling and educational psychologists (Table S1:14, 24, 25, 32, 35, 36, 43). By not acknowledging or engaging with the impact of cuts on services, the government ignores a significant contributory factor for the increased pressure on MH services and long waiting times (Table S1:24, 38, 39, 52, 55). Instead, the Green Paper [2] and the government’s response to the consultation [45] maintain that ‘the problem’ is variable quality of provision in different areas, a lack of ‘joined-up working’, and that children and young people are accessing specialist services for mild and moderate mental health problems that may be better addressed in other settings.

The responses to the Green Paper questioned whether the capacity of the Designated Senior Leads will be sufficient due to already overstretched teacher workload (Table S1:10, 25, 36, 44, 50, 55). There is an assumption that the senior lead role and the whole school approach can be successfully implemented and carried out using only training incentives rather than increased long-term funding for dedicated senior lead roles. Concerns were also raised about the appropriateness of addressing mild to moderate mental health problems in non-clinical settings and whether non-clinical staff can be as effective in prevention and early intervention (Table S1:14, 23, 32). While a greater school focus on mental health is welcomed, the Green Paper is criticised for not integrating this in a wider and more ambitious strategy that responds to the rising demand for mental health support for children and young people (Table S1:23, 32, 35, 36, 43, 46, 48) or by addressing workforce shortages and high staff turnover in CAMHS (Table S1:7, 8, 9, 14, 23, 36) and youth work (Table S1:29). Moreover, stakeholders have questioned the logic that Designated Senior Leads and mental health support teams will result in fewer and more appropriate referrals as the expansion of support in schools is likely to result in greater identification of need for specialist support (9). Furthermore, there is a risk that without the proper funding and extra staffing the new waiting time standard may result in fewer successful referrals and more limited support (Table S1:1, 8, 27, 33, 46, 48, 49).

Despite that claim that one of the ‘burning injustices of our time’ ([2] , p.3) is young people facing unequal life chances as a result of mental health conditions, the consultation highlights that the Green Paper illustrates a very narrow framing of the links between inequalities and mental health and wellbeing. The focus is on mental health problems exacerbating or leading to inequalities, with limited recognition of inequalities as a potential causal factor in mental health problems and no discussion of the role of poverty in mental health prevalence (Table S1:2, 8, 9, 13, 16, 29, 31, 32, 43, 46, 48). Further, the Green Paper highlights families as both crucial to understanding the mental health of children and young people and in responding to mental health problems. For example, the Green Paper ([2], p.32) states that, ‘good inter-parental relationships are another protective factor for child mental health, particularly for children living in poverty.’ However, the implication that appropriate parenting can protect against mental health problems reflects a limited understanding of the social determinants of health (Table S1:9, 12, 17), which would address the significance of stressors and pressures outside the family that can result in mental health problems across a family (Table S1:2, 8, 9, 11, 13, 17). The government ([45] , p.16) responded to these criticisms by explicitly recognising that disadvantage can exacerbate mental health problems, and referenced investment in the Troubled Families Programme as a response to working with ‘the whole family to overcome their multiple and complex problems’. However, this programme has been heavily criticised for its framing of certain families as ‘troubled’, thereby individualising and reducing what are much wider societal problems to a selection of ‘problem’ families [47, 48].

Stakeholders broadly agreed that schools are an appropriate setting for early intervention and provision of mental health support to children, highlighting that a school setting may work better for some children as a familiar setting without the discomfort or stigma that may be felt in some clinical settings (Table S1:12, 40, 47, 49). However, some children will experience access barriers to non-clinical settings and not all children are able to access support in schools in the same way. For example, there may be particular barriers for the 48,000 (and growing) number of children who are outside of mainstream education who are more vulnerable to mental health problems (Table S1:9, 29, 34, 37). 2 highlight that schools in more deprived areas will have higher demands. In response to concerns raised about the Green Paper’s school focus, the government’s official consultation response ([45], p6) stated

‘*we are committed to ensuring that the Mental Health Support Teams reach those most in need of the support, and are accessible to all, including those not in mainstream education and in independent schools’*

However, the response provides little detail about how barriers to accessing services for children outside mainstream education will be overcome, other than signalling that the trailblazer approach (discussed further below) will address this.

Further access concerns were raised for different groups of children perceived to be overlooked in the policy (Table S1:3, 8, 9, 11, 12, 16, 19, 20, 30, 37). Responses highlighted that some children are more vulnerable to mental health problems, such as young carers, refugee and asylum-seeking families, disabled, LGBTQ+ and looked-after children, some BAME communities and international students and that there needs to be greater focus on specific experiences and barriers faced within child and adolescent health systems (Table S1:3, 8, 12, 30). In terms of race and ethnicity, the Green Paper highlights that white children are more likely to experience a mental health disorder than black children and that both white and black children are much more at risk than Indian children [2]. However, the Green Paper ignores the complexity of cultural differences and disparities in access to treatment and experiences with mental health services (as seen in the Public Health England [49] report on ethnic inequalities in health) in its recommendations. For example, children with disabilities and special educational needs may need support for access, communication and interaction with services for the policy to be successful (Table S1:19). The lack of engagement with different access needs and ‘looked after children’, specifically, in the Green Paper has led to concerns about the extent to which their access requirements and specific circumstances are accounted for (Table S1:37). The need to engage children and young people more generally in the consultation process was highlighted (Table S1:15) to ensure that the issues that affect them most are understood and how they experience mental health service provision and need. Stakeholders recommend early intervention and prevention that is based on ‘proportionate universalism’ to cater for all children but targeted to need through proactive case finding, for example for children living in poverty, in order to reduce inequalities (Table S1:4, 15, 18 20, 24, 44). Overall stakeholder responses were critical of Green Paper’s limited engagement with the importance of inequality, predominantly highlighting that mental health support should directly address inequalities and should be more varied and accessible.

A trailblazer approach (policy implementation in a selection of sites, before wider roll out) was chosen for implementation of mental health support teams and trialling the 4-week waiting time standard. The benefits of this approach stated in the Green Paper include: identifying local differences in need, provision, and structures which will impact on the implementation of the new proposals; sharing learning about implementation from trailblazer sites with other localities; and addressing and ironing out concerns identified in the implementation of planned policy changes. The use of a trailblazer approach, however, has been criticised by stakeholders for not being ambitious enough given the severity of need and gaps in mental health service provision for children and young people, as it limits any potential benefits of the policy to no more than a quarter of children in 5 years ([5]; Table S1:5, 6, 8, 17, 21, 24, 26, 30, 35, 41, 42, 45, 49, 52, 53, 54). The potential to increase inequalities by providing support in only a select number of trailblazer areas was also criticised in the discourse about the Green Paper ([5]; Table S1:6, 7, 22, 24, 26, 28, 32, 38, 50, 55).

## Discussion: the effects of policy discourse on policy pathways

Our findings suggest the Green Paper develops a generally linear relationship between mental health causing inequality which obfuscates the role of inequality as a causal factor in understanding mental ill-health (despite evidence from stakeholders), placing greater responsibility on the individual. The policy fits an established wider trend of government’s lacking investment in promotion of good mental health [35]. Instead, UK government policy since 2010 has been focused on mental health ‘problems’ and individual responsibility rather than improving mental health for all as the policy focuses on responding to mental ill-health (crises) and inadequacies in mental health care provision rather than promoting positive mental health for all children [28]. Stakeholder responses highlighted that efforts must be made to ensure mental health support is more varied and accessible than is currently the case. For the policy to adequately respond to inequality, it must acknowledge the importance of access and there must be a recognition of current barriers and a commitment to removing them [50].

### Absences as evidence

Marmot et al. ([17] , p.5) argue that ‘austerity has adversely affected the social determinants that impact on health in the short, medium and long term’, yet the policy does not engage with austerity and budget cuts at all. Instead, it focusses on, and thus the ‘problem’ is represented to be, the way services are organised while stakeholder responses considered issues with joined up working alongside: the wider structural inequalities contributing to a mental health crisis among children and young people, barriers to access, lack of capacity for support within and outside of the NHS, and cuts to services that previously provided vital support. Furthermore, concerns were raised by stakeholders about capacity to meet need (potentially further exacerbating inequality) and the limited engagement with the complexities of needs (that accessibility and need may look different for different groups of children) are not addressed sufficiently in the policy. For Cox & Macdonald [51] the policy proposals lack an understanding of the significance of ‘culture, diversity and difference’ which is necessary to adequately support the mental health of all children and young people.

In this respect, the Green Paper fails to engage with the strong and well-established body of evidence on the social determinants of health that emphasises the multidimensional aspects of inequality (despite claims that the policy will address the ‘burning injustices’ of health inequality ([2], p.3). In short, both the structural nature of socio-economic inequalities in health and the different types of children likely to be affected by mental health problems are identified by stakeholders as generally absent from the Government’s framing and contextualisation of the policy pathways. Thus, our analysis of the Green Paper and consultation process suggests that the social determinants have been at best marginalised in the evidence-base for the policy. Instead, the Green Paper tends to focus on families as the cause of mental health problems rather than identifying the ‘causes of the causes’ [16] of health and health inequalities as factors in mental health inequalities among children and young people.

The Green Paper’s emphasis on cost (unemployment benefits and working days lost due to poor mental health) as a key driver for reducing mental health problems in children, and a continued refusal to engage with the impact of austerity and cuts on the child mental health system throughout the consultation process reflect a continued commitment to the programme of austerity. We propose that problem representations critique in this paper stem from an active disengagement with parts of the social determinants of health evidence base that are less politically and ideologically palatable for the current UK government. This is sadly a persistent theme in literature about health inequalities. Indeed, Doyal with Pennell [13] argued more than 40 years ago, that focusing on the individual origin of disease obscures the social and economic causes of ill health and is, therefore, one way of diffusing the political significance of the ‘destruction of health’. Political knowledge is therefore prioritised over established scientific and practical implementation knowledge [40], justified through particular problem representations [1, 52].

### A counter narrative illustrated through stakeholder evidence

Using Bacchi’s [1, 52] framing of ‘problems’ being discursively created and justified using specific types of politically motivated evidence [40] (Head, 2008) we identified a counter narrative, to that put forward by the Green Paper, by stakeholders with interest and expertise in child mental health that was side-lined by the government’s narrative. Society as a whole has a role to play in demanding an end to the conditions causing health inequalities [14, 16, 17, 26]. To do so, Scambler and Scambler’s [26] argue there is a need to critique and challenge the mechanisms in society that give rise to and sustain inequalities in power and wealth to address the inequalities in health and health care. Yet, our analysis of the Green Paper and consultation has demonstrated limited engagement with, and narrow framing of, inequality in relation to child mental health.

Through engagement with the wider discourses on inequalities in the mental health system, it seems stakeholders were much more aligned with a social determinants of health perspective and illustrate gaps in the evidence base of the policy. Our findings and analysis of the responses to the ‘Transforming Children and Young People’s Mental Health Provision’ Green Paper [2] from experts and advocates for child mental health illustrate the need for greater commitment to challenging the inequalities present in the children’s health systems. This commitment must take account of the interconnections between social determinants, including identity and place, and how these factors interact with access to, and experience of, mental health support [1, 16, 17, 20].

Integrating macro-level structural forces into system-wide approaches are, therefore, key in conceptualising and addressing the full range of interrelationships identified in a social determinants of health perspective. Strategies to tackle health inequalities may, therefore, be more successful when integrating wider aims such as redistribution of wealth via increased taxation or labour market regulation to address health inequalities related to poverty [14]. Yet, such macro-level sector wide policies which aim to address health inequalities are rare [53] and for Soroka & Wlezien, [54] redistribution has relatively low public support generally, making a policy intervention from national government less likely.

The localisation of health spending and responsibility [16] presents opportunities to address some of the gaps in the national policy process highlighted in this review through local policy and strategy. Where national government leaves gaps in acknowledgement of the social determinants of health and targeting health inequalities at a local level, local policy makers may be more able to address health inequalities in an embedded and systemwide way [41]. Understanding the complexity of inequalities and access needs with specific attention to place may be harnessed at a local policy level, where mental health support is available for all, but resourcing and delivery of support is targeted based on need. Local policy makers have a critical role in translating and adapting national policy for their communities, and ‘making good’ any absences and oversights in national policy relation to health inequalities. We provide evidence in this paper that local policy makers are already mindful of this task but, given the scale and complexity of this work, high-quality support (free of charge) is needed for local teams to engage with.

## Conclusions

In light of our findings we argue that ‘transforming’ children and young people’s mental health provision is imperative to respond to the growing prevalence of mental health problems and inequalities in the context of changes in policy and service provision in recent years and the impact of the Covid-19 pandemic. While the government has set out plans for addressing increasing mental health provision in a number of ways, we argue that the approach is limited by significant absences and narrow framings of inequality. We suggest that the green paper may have had a stronger focus on the social determinants of child mental health if the stakeholder responses to the extensive consultation had been more meaningfully addressed. The illustration of how policy discourse frames and produces ‘problems’ and the way evidence used (and evidence not used) is justified politically, for example the continued implicit upholding of the necessity of austerity without austerity being explicitly discussed, may be of interest to all those hoping to critique and influence policy approaches to health inequalities, not just in the context of English policy.

Our novel approach illustrates how using methods from political and social science disciplines can reveal new insights about health policies. Using a WPR approach to evaluate policy pathways with discourse analysis of policy documents and stakeholder responses highlights the mechanisms through which policies plans are constituted and justified. Particular narratives, choices in evidence bases and highlighted absences produce particular problem representations which lead to (and justify) particular policy approaches. By engaging with wider policy and health system responses, counter narratives become evident, through which alternative policy pathways can be identified.

Our research speaks to both national and local policy makers and advisors as the effects of problem representation and the complexity and importance of the social determinants of health to understanding child mental health illustrate that health inequalities must be a central focus of health policy, embedded in the realities of health systems. One of the consistent stand-out responses from the stakeholders who took part in the consultation - the ‘elephant in the room’ – was around austerity. One of the major challenges for those responsible for implementing local policy is the realities of over-stretched budgets and workforces making changes difficult to achieve. We suggest the new child health system in England, which involves Integrated Care Systems and Sustainability and Transformation Partnerships, provides an opportunity to better tackle mental health problems and inequalities in children and young people.

## Supplementary Information

**Additional file 1: Table S1.** Data extraction: Responses from online searches within one month of key dates.

## Data Availability

The qualitative data extracted and analysed during the current study is not publicly available but can be discussed or made available from the corresponding author on reasonable request. All documents analysed are publicly available and referenced in this article.
