# Supplementary material for: An analysis of English national policy approaches to health inequalities: ‘transforming children and young people’s mental health provision’ and its consultation process
Source: BMC Public Health. 2022 May 31;22:1084. doi: 10.1186/s12889-022-13473-6 (PMC9153869; doi:10.1186/s12889-022-13473-6)
Supplement: Supplementary file 1 — Additional file 1: Table S1. Data extraction: Responses from online searches within one month of key dates. [file 12889_2022_13473_MOESM1_ESM.docx]

**Table S1: Data extraction: Responses from online searches within one month of key dates**

| Data extraction: Responses from online searches within one month of key dates | | |
| --- | --- | --- |
| Sector | Search period (within one month of key date) | Author organisation/stakeholder (with date of article/response dd.mm.yy) |
| Not-for-Profit & Voluntary and Community sector | a (release of Transforming Children and Young People’s Mental Health Provision Green Paper (Department of Health & Social Care (DHSC) & Department for Education (DfE), 04.12.17) | 1. Association for Child and Adolescent Mental Health, 28.11.17) 2. Mental Health Foundation (04.12.17) 3. Student Minds (05.12.17) 4. The Kings Fund (08.12.17) 5. Anti-bullying Alliance (19.12.17) |
|  | b (consultation deadline 02.03.18) | 1. Royal Society for the encouragement of Arts, Manufactures and Commerce (09.02.18) 2. Royal College of Psychiatrists Consultation (01.03.18) 3. The Kings Fund (02.03.18) 4. The Children’s Society (02.03.18) 5. Become (March 2018) 6. Children's Rights Alliance for England (March 2018) 7. Centre for Mental Health (March 2018) 8. Children and Young People’s Mental Health Coalition (March, 2018) 9. BEAT eating disorders, (March, 2018) 10. The Big Lottery Fund (March, 2018) 11. Young People’s Health (March, 2018) 12. Youth Access (March, 2018) 13. Royal College of Nursing (06.03.18) 14. Place2be (08.03.18) 15. Royal College of Paediatrics and Child Health (9.3.18) 16. Universities UK (18.03.18) 17. Nurture UK (20.03.18) |
|  | c (release of ‘Failing a generation’ report (Education and Health and Social Care Committees, 09.05.18) | 1. British Association for Counselling and Psychotherapy (09.05.18) |
|  | d (release of ‘Government Response to the Consultation’ (DHSC & DfE, 25.07.18) and ‘Government Response to the ‘Failing a generation’ report (DHSC & DfE, 25.07.18) | 1. CYP Now, (24.07.18) 2. National Children’s Bureau (25.07.18) 3. National Association Head Teachers (25.07.18) 4. British Association for Counselling and Psychotherapy (26.07.18) 5. Young Minds (26.07.18) 6. National Youth Agency (27.07.18) 7. National Society for the Prevention of Cruelty to Children (28.07.18) 8. Schools in Mind (03.08.18) |
| Professional Associations & TUs | a | 1. The British Psychological Society (05.12.17) 2. NHS Confederation (04.12.17) |
|  | b | 1. Association of Directors of Child Services (01.03.18) 2. Unite the Union (02.03.18) 3. Association of School and College Leaders (02.03.18) |
|  | c | 1. British Association of Social Work (11.05.18) |
|  | d | 1. Local Government Association (25.07.18) 2. NHS Providers (25.07.18) |
| Private sector | a | 1. Ellern Mede Eating Disorder Services (04.12.17) |
| Education and training Organisations | a | 1. Schools Week (03.12.17) 2. Schools Week (03.12.17) 3. Social, Emotional and Mental Health (10.12.17) |
|  | b | none |
|  | c | 1. Cache (17.05.18) |
|  | d | 1. Schools Week (25.07.18) |
| Media | a | 1. The Guardian (23.11.17) 2. BBC (03.12.17) 3. The Guardian (03.12.17) 4. Huffington Post (07.12.17) |
|  | b | none |
|  | c | 1. BBC (09.05.18) 2. Early Years Alliance (09.05.18) |
|  | d | 1. The Independent, (25.07.18) 2. Cover Magazine (26.07.18) 3. Health Service Journal (26.07.18) 4. Happiful (27.07.18) |
